# Intermittent Energy Restriction, Weight Loss and Cardiometabolic Risk: A Critical Appraisal of Evidence in Humans

**DOI:** 10.3390/healthcare9050495

**Published:** 2021-04-22

**Authors:** Alexia L. Katsarou, Nicholas L. Katsilambros, Chrysi C. Koliaki

**Affiliations:** 1 Third Surgical Clinic, Hygeia Hospital, 15123 Athens, Greece; katsaroualexia@gmail.com; 2 Research Laboratory Christeas Hall, Medical Faculty, National Kapodistrian University of Athens, 11527 Athens, Greece; nicholaskatsilambros@gmail.com; 3 First Propaedeutic Department of Internal Medicine and Diabetes Center, Laiko University Hospital, National Kapodistrian University of Athens, 11527 Athens, Greece

**Keywords:** intermittent energy restriction, periodic fasting, alternate day-fasting, time-restricted feeding, obesity, diabetes, cardiometabolic risk

## Abstract

Dietary patterns with intermittent energy restriction (IER) have been proposed as an attractive alternative to continuous energy restriction (CER) for the management of obesity and its associated comorbidities. The most widely studied regimens of IER comprise energy restriction on two days per week (5:2), alternate-day energy restriction by 60–70% (ADF), and timely restriction of energy intake during a specific time window within the day (TRF; time-restricted feeding). Although there is some evidence to suggest that IER can exert beneficial effects on human cardiometabolic health, yet is apparently not superior compared to CER, there are still some critical issues/questions that warrant further investigation: (i) high-quality robust scientific evidence regarding the long-term effects of IER (safety, efficacy, compliance) is limited since the vast majority of intervention studies had a duration of less than 6 months; (ii) whether the positive effects of IER are independent of or actually mediated by weight loss remains elusive; (iii) it remains unknown whether IER protocols are a safe recommendation for the general population; (iv) data concerning the impact of IER on ectopic fat stores, fat-free mass, insulin resistance and metabolic flexibility are inconclusive; (v) the cost-effectiveness of IER dietary regimens has not been adequately addressed; (vi) direct head-to-head studies comparing different IER patterns with variable macronutrient composition in terms of safety and efficacy are scarce; and (vii) evidence is limited with regard to the efficacy of IER in specific populations, including males, the elderly and patients with morbid obesity and diabetes mellitus. Until more solid evidence is available, individualization and critical perspective are definitely warranted to determine which patients might benefit the most from an IER intervention, depending on their personality traits and most importantly comorbid health conditions.

## 1. Introduction

Identifying effective strategies for long-term weight control is critical to reduce the alarming prevalence of obesity worldwide and mitigate obesity-associated health risks [1,2]. Excess energy intake and a sustained positive energy balance are unequivocally associated with increased cardiometabolic morbidity and mortality [3]. A solid body of clinical and experimental evidence has clearly demonstrated that a negative energy balance achieved by reduced caloric intake may confer substantial cardiometabolic and overall health benefits in animals and humans [3,4]. Caloric restriction has been shown to prevent several degenerative diseases such as malignancies, cardiovascular disease (CVD), type 2 diabetes mellitus (T2DM) and dementia; slow down the progression of age-dependent functional decline; and most importantly, extend lifespan and promote longevity in rodents and non-human primates [5,6,7,8,9].

The vast majority of studies on weight loss have investigated the efficacy of conventional hypocaloric diets providing continuous energy restriction (CER) [10,11]. Over the past decade, a novel model of dietary intervention has attracted increasing scientific attention, based on the intermittent restriction of caloric intake with shifts between periods of reduced caloric intake and periods of unrestricted ad libitum feeding (IER; intermittent energy restriction) [12]. Dietary patterns with IER have emerged as an attractive alternative to CER for the management of obesity and its associated comorbidities mainly for two reasons: first, IER requires caloric restriction only on prespecified days of the week or for relatively prolonged intervals during each day, which is theoretically more feasible compared to the conventional approach of daily caloric restriction, which is known to be associated with poor compliance [13]; and second, some of the metabolic benefits achieved by caloric restriction are primarily related to energy deficit *per se* rather than weight loss, and are thus attenuated upon cessation of the negative energy balance [14]. Based on the above rationale, it has been suggested that repeated short periods of significant caloric restriction on specific weekdays may help overweight/obese individuals lose weight and preserve the accomplished metabolic benefits even in periods of liberal feeding [15]. The most extensively studied regimens of IER comprise energy restriction on two days per week (5:2), alternate day energy restriction by 60–70% and timely restriction of energy intake during a specific time window within the day (TRF; time-restricted feeding) [4,15,16,17]. IER has produced a broad spectrum of beneficial health effects in animal models of chronic diseases [7,8,9], although high-quality research in humans has been limited.

The aim of the present review is to describe the most frequently used versions of IER in clinical research settings and elucidate the health claims of this eating pattern in relation to cardiometabolic risk factor modulation. More specifically, the effects of IER on body weight, lipid profile, hypertension and glucose metabolism are presented, and a number of important concerns, limitations, knowledge gaps and unresolved questions related to IER are critically discussed. The studies reviewed herein have been performed in adults, and not in children or adolescents.

## 2. Different Patterns of IER 

As already mentioned, one of the most frequently applied patterns of IER is the alternate-day fasting (ADF) protocol, according to which energy consumption is usually reduced by 75% for three or four non-consecutive days of the week [18]. On “free” weekdays, energy consumption varies from 1500 to 2500 kcal for women and 2500 to 3500 kcal for men. The other version of IER is the 5:2 protocol, also termed periodic fasting, according to which energy consumption reaches 500–600 kcal (sometimes even zero kcal) during each of the two non-consecutive weekdays of energy restriction, while during the remaining five weekdays energy consumption returns to usual [4,17]. The third IER regimen comprises the 16:8, 18:6 and 20:4 TRF protocols, according to which there is a daily 8-, 6- or 4-h eating window for energy consumption and a 16-, 18- or 20-h window of fasting, respectively [17,19,20,21]. The present review is mainly based on the first two regimens, since comparative data on TRF are still not enough to allow drawing safe conclusions. 

## 3. Effects of IER on Weight Loss and Maintenance

To summarize the available evidence, it has been shown that IER can lead to a weight loss of 4–10% within a time period of 4–24 weeks, depending on the dietary pattern [22,23,24,25,26,27,28,29,30]. In particular, ADF has been associated with a mean weight loss of 0.75 kg per week, whereas IER 5:2 (periodic fasting) has been associated with a mean weight loss of 0.25 kg per week, apparently due to its less stringent weekly negative energy balance [31,32]. The critical question to address is whether this weight loss efficacy is superior to that observed after CER. The majority of clinical studies in this field failed to demonstrate any statistically significant differences in weight loss efficacy between IER and CER. In further detail, intermittent dietary interventions with caloric restriction by 55–70% for 2 days per week [33,34,35], 50% for 4 days per week [36], 10–70% for 3–7 days per week [37], ADF with caloric restriction of 70% [38] and 16:8 TRF for 12 weeks [39], have led to comparable weight loss with isoenergetic continuous hypocaloric diets or consistent meal timing in overweight and obese individuals. According to two systematic reviews and meta-analyses investigating the effects of IER interventions of at least 6 months duration on weight loss, IER appears to be superior to the absence of any dietary intervention in terms of weight loss and is thus proposed as an effective approach for the treatment of obesity [40,41]. However, both meta-analyses emphasized that the observed effects of IER were not superior to those of CER [40,41]. A 12-month randomized clinical trial (RCT) aiming to investigate the effects of ADF on weight loss and maintenance in metabolically healthy obese adults concluded that ADF does not produce greater adherence, weight loss efficacy or weight loss maintenance than CER [42]. In this study, participants were randomized to ADF, CER or a control intervention for one year. The trial involved a 6-month weight-loss phase, followed by another 6-month weight loss maintenance phase. Of note, participants in the ADF group tended to consume more calories than suggested during the fast days and less during the “feast” days, while participants in the CER group managed to comply successfully with the prescribed energy regimen [42].

Weight loss maintenance and prevention of weight regain represent an extremely important component of a successful weight loss intervention and depend to a great extent on the self-discipline and motivation of the dieters. Predictors of successful long-term weight maintenance after initial weight loss involve frequent self-monitoring of body weight [43], medical supervision for psychological support [44] and high levels of physical activity [45]. In conventional hypocaloric diets, weight loss maintenance is defined as maintaining a weight loss of at least 10% of the initial body weight for at least 12 months [46]. Overall, IER diets have shown an adequate weight loss maintenance capacity in the short term (3–6 months) [33]. However, evidence regarding the long-term potential of these diets to achieve weight loss maintenance is limited, and more clinical data to address this issue are needed. IER-related factors that could unfavorably affect weight loss maintenance by compromising compliance include the feeling of hunger experienced by some participants, which could potentially promote an increased caloric intake on the days of unrestricted feeding [47] and the difficulty to carry out the activities of daily living, especially in the setting of ADF [48].

Under conditions of profound caloric restriction, the enhanced susceptibility to the lipolytic effects of catecholamines promotes an elevated rate of splanchnic lipolysis and an increased free fatty acid (FFA) flux [48]. In support of this mechanism, studies in overweight subjects [49,50] and patients with T2DM [50] have shown that hypocaloric diets with drastic caloric restriction of 50–70% can lead to significant reductions of total and hepatic fat. IER diets can also promote fat mass loss [23,33,51]. Considering that IER diets induce a more pronounced mobilization of FFA compared to CER [52,53,54], it would be rationally expected that these diets can lead to similar or even superior loss of adipose tissue. In line with this assumption, an IER diet with low carbohydrate intake has been associated with a greater reduction of fat mass compared to an isoenergetic continuous hypocaloric diet in overweight women [33].

With regard to lean body mass, it is generally accepted that conventional hypocaloric diets reduce skeletal muscle mass by 10–60% of total weight loss achieved [55]. This reduction appears to be lower with IER diets [25,38,56]. Although IER is considered to be more effective in muscle mass preservation than CER, not all studies corroborate this statement [57]. The efficacy of IER in skeletal muscle mass preservation may be significantly enhanced by its combination with high dietary protein intake [58] and physical activity [37,55,59]. It is also affected by the degree of adiposity and the % restriction of caloric intake [55]. It has been proposed that consuming 1.2 g/kg body weight of protein in the setting of IER is more efficient in preserving skeletal muscle mass than IER regimens with lower protein intake [33]. It has been also suggested that isometric resistance training can not only improve muscle tolerance but also preserve skeletal muscle mass despite weight loss [23,60].

## 4. Effects of IER on Glucose Metabolism and Insulin Sensitivity

Studies in overweight and obese individuals investigating the impact of IER upon glycemic profile and insulin sensitivity assessed by a variety of methods have produced heterogeneous and inconsistent findings. In some of these, IER had no effects on blood glucose and/or insulin levels in non-diabetic subjects. More analytically, no statistically significant differences were observed between IER and CER followed for 4–24 weeks, either in terms of fasting glucose [26,27,30,33,61,62] or glycated hemoglobin HbA1c levels [33]. An IER dietary regimen with caloric restriction of 55–70% on two days per week, with low carbohydrate intake and ad libitum intake of protein and monounsaturated fatty acids, has been associated with a greater reduction in fasting insulinemia and Homeostasis Model Assessment Index for Insulin Resistance (HOMA-IR) compared to a conventional hypocaloric diet, whereas insulin sensitivity assessed by an intravenous glucose tolerance test remained unchanged [22]. Additional studies comparing IER with CER in overweight and obese non-diabetic women have further corroborated the previous findings of reduced fasting insulin levels and HOMA-IR after 3–6 months of IER [38]. Of note, HOMA-IR index, reflecting mainly hepatic insulin resistance, was more significantly reduced after IER compared to CER, either measured after the period of fasting or the period of unrestricted feeding [34]. A review and meta-analysis summarizing the effects of IER on glucose metabolism based on 12 intervention studies of at least one-month duration in a total of 545 participants concluded that fasting glucose and HOMA-IR are significantly reduced after IER [63].

It has been shown that both insulin-mediated peripheral glucose disposal (skeletal muscle insulin sensitivity) and insulin-induced suppression of lipolysis (adipose tissue insulin sensitivity) during an euglycemic hyperinsulinemic clamp are significantly ameliorated after 2 weeks of IER in healthy subjects. This improvement has been mainly attributed to the increased circulating adiponectin concentrations observed after 20 h of fasting [64]. Gender appears to be a significant determinant of the impact of IER upon insulin sensitivity. A study has shown that male participants displayed reduced postprandial insulin levels, improved glucose tolerance and enhanced insulin sensitivity after 3 weeks of IER, as opposed to female participants, who presented impaired glucose tolerance and increased skeletal muscle insulin resistance after the same intervention, without any negative effects on endogenous insulin secretion [47]. It should be noted, however, that measurements were performed after 36 h of fasting in this study, and thus, the adverse effects on insulin sensitivity might be partly related to the accentuated metabolic fluctuations and FFA fluxes induced by prolonged fasting. Gender-specific beneficial effects of IER on insulin sensitivity have been also reported in male subjects with prediabetes who experienced an improvement in pancreatic β-cell responsiveness and insulin sensitivity after 5 weeks of an early TRF intervention (6-h feeding period with dinner before 3 p.m.) even in the absence of weight loss [65]. It is important to note that the studies presented above investigating the effects of IER on glucose metabolism and insulin sensitivity were focused on IER and performed no direct comparison with CER [47,64,65]. In non-obese subjects, IER appears to lead to differential effects on peripheral insulin resistance compared to overweight/obese (negative vs. positive). At present, the precise consequences of repeated short-lived elevations of circulating FFA levels in terms of hepatic and skeletal muscle insulin resistance are inadequately characterized and warrant further investigation. These effects might be particularly meaningful for cohorts with increased vulnerability to FFA elevations and the resultant lipotoxicity, such as female and normal-weight individuals [66].

Data of comparative evaluation of IER vs. CER in terms of improving glycemic control in overweight and obese patients with T2DM are limited. In this field, it has been shown that an IER diet with caloric restriction of 70% on 4 days per week for at least 12 weeks did not result in significant alterations of HbA1c levels in overweight and obese T2DM patients compared to a continuous hypocaloric diet [36]. Similar HbA1c reduction after IER and CER in patients with T2DM has been reported in other studies as well [35]. It has been also demonstrated that HbA1c reduction is positively correlated with % reduction of total and visceral fat mass [35]. A combination of a hypocaloric diet with 25% energy deficit with alternating periods of IER has been associated with significant beneficial effects on HbA1c in T2DM patients and has thus been proposed as an effective dietary strategy to optimize glycemic control in diabetic patients [67].

## 5. Effects of IER on Lipid Profile

The majority of human studies have shown a beneficial effect of IER diets on the lipidemic profile of the participants [30,37,38,51], although there have also been studies that failed to demonstrate any significant differences in lipid levels between IER and CER [29,33,34,36]. In more detail, IER applied for 3–24 weeks has been associated with reduced total cholesterol levels by 6–21%, reduced fasting triglycerides by 16–24% and reduced LDL cholesterol levels by 7–32% [37,38,51]. Interestingly, it has been shown that a dietary intervention with intermittent caloric restriction of at least 70% was associated with a shift from small dense to larger and less atherogenic LDL particle fractions [59,60]. Of note, the aforementioned dietary intervention has also been associated with an improved lipid profile in patients with T2DM [36]. Additional studies have further confirmed the beneficial effects of IER on lipidemic profile, without, however, clarifying whether the observed effects are related to the dietary intervention per se or rather to the concomitant weight loss and energy deficit [61]. Another study investigating the effects of a TRF intervention, whereby participants abstained from food intake for more than 12 h per day and consumed only an evening meal, has reported both pro-atherogenic (increased LDL) and anti-atherogenic (increased HDL, reduced triglycerides) lipid profile alterations after 8 weeks of intervention [68]. With regard to the ectopic deposition of triglycerides in non-adipose tissues, reflecting visceral fat accumulation, fasting for 24–48 h resulted in elevated intramyocellular triglycerides in non-obese women [69] and increased intrahepatic triglycerides in non-obese men [69,70].

A recent systematic review and meta-analysis investigating the effects of IER and CER on lipid profile concluded that both dietary approaches reduce the levels of total cholesterol, LDL cholesterol and triglycerides but have no effect on HDL cholesterol [71]. In fact, CER was associated with greater reductions in total cholesterol levels compared to IER (−10.3 mg/dL vs. −3 mg/dL, respectively), and greater reductions in lipid markers were related to higher baseline (pre-intervention) levels. Of note, when caloric reduction was greater than 50%, no significant reductions in total and LDL cholesterol levels were observed [71]. In the 12-month RCT included in this meta-analysis, no differences were found between intervention groups regarding total cholesterol and triglyceride levels [42]. HDL cholesterol was 6.2 mg/dL higher in the IER group compared to CER at 6 months of intervention, but this difference lost significance at 12 months [42]. Furthermore, LDL cholesterol levels were elevated by 11.5 mg/dL at 12 months in the IER group compared to CER [42]. Of note, no effects of IER interventions on non-HDL cholesterol, which is also included in cardiovascular risk assessment recommendations, have been reported in the studies mentioned above. 

## 6. Effects of IER on Blood Pressure 

Several studies report comparable blood pressure (BP) reduction with IER and CER diets [33,34,37]. Some other studies suggest beneficial effects of IER on BP levels. A small study in men with prediabetes has shown an average reduction of systolic BP by 11 ± 4 and diastolic BP by 10 ± 4 mm Hg after 5 weeks of a TRF dietary intervention with fasting for 18 h periods [65]. In patients with T2DM, IER has been found to be more effective in lowering diastolic BP compared to CER [35]. Furthermore, an IER diet with caloric restriction on 5 consecutive days per month for 3 months has been shown to reduce both systolic and diastolic BP in healthy individuals [30]. Of note, IER interventions of 3–24 weeks duration have led to BP reductions only if at least 6% weight loss could be achieved. Systolic BP reductions associated with IER range between 3 and 8%, and diastolic BP reductions range between 6 and 10%. These data have been primarily shown in participants with pre-hypertension [31,32].

A systematic review and meta-analysis investigating the effects of IER on BP levels in a total population of 1400 participants concluded that both systolic (−3.3 mm Hg) and diastolic (−1.6 mm Hg) BP can be significantly lowered with IER [72]. It should be mentioned, however, that interventions lasting less than 12 weeks were found to be more effective than longer-lasting interventions, which could imply that compliance with IER might be gradually fading and probably cannot be maintained for a long time, thus raising long-term efficacy concerns.

## 7. Effects of IER on Markers of Subclinical Inflammation

IER has been associated with a beneficial effect on circulating inflammatory markers in humans [33,60,73]. To our knowledge, only one study has systematically reviewed RCTs regarding the effects of IER on plasma concentrations of inflammatory biomarkers implicated in the pathogenesis of atherosclerosis and CVD complications [63]. In total, 18 RCTs were included in a total of 700 participants. Although no significant improvements were observed in levels of interleukin-6 (IL-6) and tumor necrosis factor a (TNFa), CRP was reduced more significantly after IER compared to energy-restricted diets, and greater reductions were achieved in caloric restriction > 50%, overweight and obese individuals and interventions lasting at least two months [63].

## 8. Proposed Mechanisms Mediating the Effects of IER

**Figure 1:**
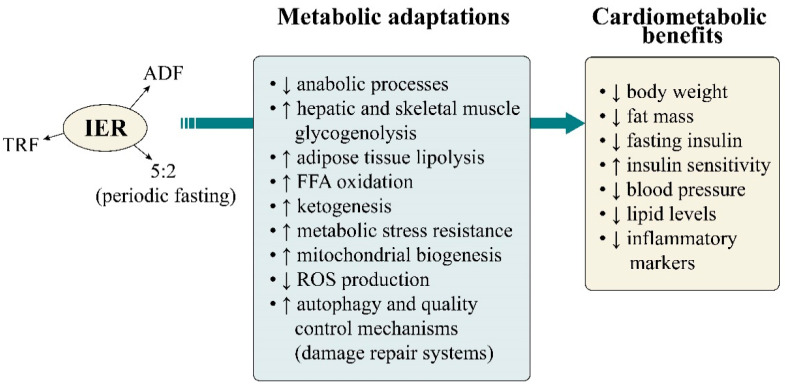
IER has been claimed to be associated with a variety of favorable metabolic adaptations leading to cardiometabolic benefits, comprising reduced anabolic processes, increased hepatic and muscle glycogenolysis, elevated FFA oxidation and ketogenesis, cellular resistance to metabolic stress, enhanced mitochondrial biogenesis, reduced reactive oxygen species (ROS) production, and increased quality control mechanisms (i.e., autophagy), ensuring disposal of damaged molecules and cellular component repair.

It has been postulated that the positive effects of IER on cardiometabolic health and beyond are partly attributed to mechanisms associated with circadian rhythms, glucose-to-ketone metabolic switching, mitochondrial function and lipid metabolism, as summarized in Figure 1 [15,21,74,75]. 

Sophisticated experimental studies have shown that when the master clock in the brain (suprachiasmatic nucleus), as well as other peripheral clocks (i.e., in liver, adipose tissue, skeletal muscle) are desynchronized, the risk of cardiovascular and metabolic dysregulation is increased [21]. In this context, energy consumption that does not keep pace with the normal hormonal rhythms of the body such as eating late at night (circadian misalignment) may disrupt energy balance and promote a variety of metabolic perturbations [21,75,76]. It is also noteworthy that TRF short-term trials have shown that the alignment of the feeding period with the circadian rhythms may result in weight loss and improvements in insulin sensitivity as well as inflammation [77].

IER is further characterized by a metabolic switch from liver-derived glucose to adipose cell-derived ketones either daily or several days per week [15]. Periods of energy restriction sufficient to cause depletion of hepatic glycogen stores trigger a metabolic switch toward the use of FFAs/ketones. Cells and organ systems adapt to this bioenergetic challenge by activating signaling pathways that up-regulate mitochondrial function, stress resistance and antioxidant defenses while up-regulating autophagy to remove damaged molecules and recycle their components [15]. IER has been associated with a great variety of favorable metabolic adaptations, comprising down-regulated anabolic processes, elevated FFA oxidation and ketogenesis, increased hepatic and skeletal muscle glycogenolysis, reduced reactive oxygen species (ROS) production, enhanced mitochondrial biogenesis, improved cell survival, reduced leptin and increased adiponectin secretion [15,16,74,75]. Interestingly, the enhanced lipid oxidation and ketogenesis induced by fasting remain active even after resumption of food intake, the effect being an improved postprandial lipid metabolism [76].

## 9. Limitations and Safety Concerns

The most important issue is that there are currently no long-term safety data with IER diets, especially in the population of normal-weight subjects.

Furthermore, in normal-weight subjects who cannot adequately control the amount of ingested food (unrestrained eaters), and especially in the setting of strict dietary interventions requiring a drastic caloric restriction (e.g., 70%), IER diets have been associated with a multitude of adverse effects including increased hunger feelings, fatigue, irritability, mood disorders, concentration difficulties and uncontrolled hyperphagia on the days of unrestricted feeding, which is frequently manifested as binge eating disorder [4]. This is not the case for overweight and obese individuals who follow IER diets.

There have been also concerns that strict and prolonged hypocaloric diets, either continuous or intermittent, may disrupt the hypothalamus-pituitary-gonadal axis of young women, promoting menstrual abnormalities and potentially interfering with their reproductive ability. Although there is currently no solid evidence to suggest that IER diets have a clinically meaningful detrimental effect on ovulation and fertility of women of reproductive age [4], it should be noted that long-term data addressing this issue are lacking.

## 10. Knowledge Gaps and Unresolved Questions

Although there is some evidence to suggest that IER can exert beneficial effects on human cardiometabolic health, yet apparently not superior compared to CER, there are still some questions that need further examination.

High-quality robust scientific evidence (based on RCTs) regarding the long-term effects of IER remains limited. The vast majority of intervention studies included in the systematic reviews and meta-analyses had a duration of less than 6 months. Of note, two RCTs of at least 12 months duration concluded that IER and CER result in similar weight loss and improve CVD risk factors to a similar extent, conveying the take-home message that IER diets (ADF and periodic fasting) are not superior to conventional CER [78,79]. An important point in this context is that feelings of hunger may limit long-term adherence to IER [79].

Whether the positive effects of IER are independent of or actually mediated by weight loss remains elusive. In other words, it is yet unclear if IER represents another weight loss regimen or rather an independent cardioprotective and health-promoting behavioral approach. Although evidence from animal studies clearly suggests that IER itself is related to metabolic improvements [80], this is not completely elucidated in human studies. Moreover, if the effects of IER are independent, it would be interesting to examine any additive effects of multi-disciplinary interventions including IER, Mediterranean or DASH diet protocols, along with psychological approaches such as cognitive behavior therapy and psychoeducation.

A critical question that needs to be answered is whether IER protocols are a safe recommendation for the general population. Possible contraindications, including psychiatric disease, food preoccupation, disordered eating and body weight regain related to the adoption of IER, are not adequately discussed in the literature [81].

Furthermore, data concerning the impact of IER diets on ectopic fat stores, adipocyte size, fat-free mass, insulin resistance and metabolic flexibility are heterogeneous and inconclusive. Some studies in normal-weight subjects have unraveled detrimental effects of intermittent diets on fat distribution and metabolic homeostasis, which raises safety concerns and warrants further investigation. Moreover, the cost-effectiveness of IER has not been adequately addressed, and direct head-to-head studies comparing different IER patterns in terms of safety and efficacy are scarce. Evidence is also limited with regard to the efficacy of IER in specific populations, including males, children, adolescents, elderly and patients with morbid obesity and T2DM. Studies investigating the effects of IER interventions with specific characteristics in terms of macronutrient composition (i.e., low-fat, low-carbohydrate, very low-carbohydrate, high-protein) are also lacking.

Thus, unanswered questions in the field comprise whether long-term compliance with IER is superior to CER, whether IER-induced weight loss can be maintained for long and what the optimal characteristics are of an IER intervention (intermittent intervals and macronutrient composition) that can lead to maximal efficacy in weight loss and cardiometabolic risk factor improvement.

## 11. Conclusions 

IER is an interesting subject in the research field of body weight regulation, CVD prevention and overall health promotion. However, given the relative paucity of long-term data, it is yet not clear whether IER provides a safe recommendation for the general population. Regarding body weight loss, parameters such as long-term adherence and compliance, personalization, efficacy, high-quality foods and balanced diet, negative energy balance, realistic goal setting and safety should be underlined.

The wide popularity of intermittent diets along with the knowledge gaps highlighted in the present review underscore the vital need for future rigorous research in the field of intermittent dieting with appropriately designed, long-term, randomized studies in several patient subgroups. Additional studies are also required in the population of children and adolescents who are also affected by the obesity pandemic. Until more solid evidence is available, individualization and critical perspectives are definitely warranted to determine which patients might benefit the most from an IER dietary intervention, depending on their personality traits and most importantly comorbid health conditions.

## Abbreviations

ADF: alternate-day fasting; FFA: free fatty acid; IER: intermittent energy restriction; ROS: reactive oxygen species; TRF: time-restricted feeding.
